# Ecologising Invited and Uninvited Patient Participation in Russia

**DOI:** 10.1111/hex.14150

**Published:** 2024-07-28

**Authors:** Vlas Nikulkin, Yan Vlasov, Olga Zvonareva

**Affiliations:** ^1^ Health, Ethics and Society Department, Care and Public Health Institute Maastricht University Maastricht The Netherlands; ^2^ Department of Neurology and Neurosurgery Samara State Medical University Samara Russia

**Keywords:** deliberation, invited participation, nondemocratic situations, patient councils, patient organisations

## Abstract

**Introduction:**

Public participation can be both supported and limited by decision‐makers. Therefore, citizens either participate in top‐down approved formats or have to turn towards subversion. These different participation practices, called invited and uninvited, are often treated by researchers as mutually exclusive. In this article, we present the case of patient organisations' involvement in various state‐controlled deliberation bodies in Russia, which does not fit into a smooth binary distinction of the patient participation practice. Instead, identified patient participation practices combine interaction approved by gatekeepers with interaction, which are subversive and grassroots‐initiated. Conceptually, it means that invited and uninvited participation can be better understood as intertwined ecologies.

**Methods:**

The article is based on a qualitative ethnographic study, which includes participatory observations of the meetings of state‐controlled public participation bodies, such as public councils, 51 semi‐structured interviews with members of these bodies and an analysis of the relevant policy and methodological documents. Informed consent to record and transcribe all interviews was obtained. Thematic analysis has been used to produce the results.

**Results:**

Russian patient organisations often work informally and independently of state‐approved practices expected from them. Some subversive practices happen outside official meetings, others become widely used best practices and others remain everyday mundane interactions, which contribute to the maintenance of the independence of patient organisations against otherwise dominating and nondemocratic state actors.

**Conclusion:**

The ecologising approach to patient participation, which interprets invited and uninvited practices as interconnected, has better explanatory power for cases in which citizens maintain independence despite all limitations associated with authoritarian settings. Conceptualising invited and uninvited practices as situations, or separate time‐ and space‐bound events, is a helpful theoretical framework for understanding diverse and seemingly contradictory public participation practices.

**Patient or Public Contribution:**

Research participants communicated amendments to the initial research framework to incorporate their needs. Repeated interviews allowed triangulation of preliminary findings with research participants. The article is co‐authored with the patient organisation representative, who has contributed directly to data analysis and presentation.

## 1 Introduction

Patient participation has grown widely in recent decades [1, 2, 3, 4]. Patients successfully contribute to healthcare provision [5], organisation [6] and governance [7]. Despite the expansion of patient participation, the credentialed expertise of medical professionals and the everyday experiences of patients are often seen as ‘intrinsically distinct’ [8, p. 2315]. As a result, credentialed experts, whose knowledge has higher authority, retain their powers. In practice, this means that patient participation is often facilitated and led from above [8, p. 2315, 9, 10, 11] and patients must be ‘invited’ to participate in decision‐making by these gatekeepers [11, 12]. Being invited, patients often perceive themselves and are treated as guests who have to follow the guidance of those who invited them. Consequently, subversion is unlikely to emerge in such top‐down organised invited participation settings [11, p. 107, 13].

For autonomy and citizen‐led healthcare governance and provision, alternative ‘uninvited’ participation formats are then needed. Some examples of such invited participation are collective action street protests and individual mundane practices of navigating the healthcare system [14]. The above distinction between invited and uninvited participation is often presented theoretically and in health practice as mutually exclusive oppositions [11, 14, 15]. Such a binary approach to participation, however, cannot explain the multiplicity and complexity of participatory practices. For example, in the same clinic, patients might follow medical advice, negotiate treatment or covertly act against medical advice given to them [16, p. 14]. Hence, the same setting includes different practices.

Furthermore, invited participation formats might be institutionally detached from actual decision‐making, while various uninvited practices might support ‘embeddedness’ of participatory practices in governance processes [17, p. 134]. Therefore, we argue that patient participation is better understood not in terms of rigid settings and binary oppositions but as situations or ‘time and space localised singular events’ that evoke particular discursive ‘repertoire’ and material practices [18, p. 18]. It means that invited and uninvited participation have to be approached as intertwined and mutually reinforcing series of events rather than incompatible forms of practice. Hence, this article contributes to conceptualisation of participation as relational and shifty practices [8, p. 2315] co‐produced by ecologies of ‘multiple, diverse, entangled and interrelating collectives’ [19, p. 16]. In support of the argument regarding intertwined invited and uninvited participation, we present the case of Russian patient organisations and their uninvited practices in and around multiple and omnipresent invited participation deliberation formats.

## 2 Materials and Methods

### 2.1 Settings

The health governance in Russia is highly centralised. The main actors are the federal and regional ministries of healthcare, the controlling body of Roszdravnadzor and the relevant committees of the legislatures. The Ministry of Labour and Social Protection Medico‐Social Expertise bureaus (MSE), the Ministry of Finances, the Ministry of Trade and the Presidential Administration are some other state actors relevant to healthcare governance. These actors are in charge of providing extensive state guarantees for citizens retained from the Soviet past [20]. The practice, however, does not always uphold formal obligations [21]. Therefore, public participation by patients, called ‘public control’ by Federal Law N212 ‘On public control’ [22] and mandated by Federal Law N323 ‘On the basics of protecting the health of citizens’ [23], is vital to ensure that healthcare is provided as designed. Key elements of the patients' public control system are various non‐binding deliberative bodies, which are discussed in detail in the next section.

### 2.2 Data Collection

This article presents the results of the qualitative ethnographic research conducted from November 2021 to February 2023. The fieldwork was primarily conducted in Moscow and five other regions by Vlas Nikulkin. The collected data include semi‐structured interviews, participatory observations and documents. Data collection has been guided by the data ‘saturation’ principle [24]. Hence, theoretical considerations and redundancy in new data produced were more important than the representativeness of a sample.

We conducted 51 semi‐structured interviews with 43 research participants from 16 patient organisations and a think tank closely affiliated with the patient community. Research participants represent 19 Russian regions. All but two interviewed research participants were members of at least one deliberation body. We obtained written informed consent via forms developed as part of the Research Ethics Committee approval process (Decision FHML‐REC/2021/109). Personal data collection was guided by a minimisation principle; hence, only key statuses, such as geographical location and job position, have been identified. All names in the article are pseudonyms.

All but three interviews were conducted online, either via Zoom or WhatsApp, primarily to mitigate the COVID‐19 virus spread risk. Interviews lasted from 30 min to 3 h, with the average time being about 1 h. The interview guide for patient organisations' members focused on informal practices used to mitigate any controlling tendencies in the invited participation formats. The other interview guide informed follow‐up interviews, focusing on changes between the first (January–April 2022) and second (October–December 2022) data collection periods.

Participatory observations include two regional patient council (officially, councils of member‐based organisations on the rights of patients, Rus. *sovety obschestvennykh organisatsiy po pravam patsientov*) meetings in Syznovo and Ivnyak, two federal public council (Rus. *obschestvennye sovety*) meetings, one annual video conference meeting of the regional patient council chairs and secretaries and a recording of the Chumakhla regional public council meeting. Participant observations also covered patient organisations' everyday work (two offline visits to the offices) and events organised by patient organisations, including seven online conferences and roundtables, and three regular working meetings of the federal‐level patient organisation's leadership.

The analysed documents account for 73 entities recorded in the document database. The recorded entities include federal and regional legislation; policy documents and governmental orders; handbooks, recommendations and reports by patient organisations; public and patient councils' regulations and minutes; patient organisations' member surveys; and patient organisations' website contents, in particular news and educational materials for members, such as ‘how to write a complaint’ guide with templates. Some additional circumstantial materials, such as posts in social media groups and patient school materials, were used in the data analysis.

### 2.3 Data Analysis

The generated data were analysed using thematic content analysis. The analysis was guided by the goal of reconstructing patient participation in healthcare governance dominated by authoritarian situations. The coding scheme was developed alongside data generation and adapted in response to the insights emerging from the fieldwork. The interviews were analysed *verbatim*. Participatory observation diary notes required an additional step of thick description [25, p. 1973]. Altogether, 61 codes and 12 code groups were identified using Atlas.ti software. Codes were primarily descriptive—that is, sourcing from the data—although some interpretative codes were also used based on the initial conceptual framework. Some examples of the codes relevant to (un)invited participation topics are ‘constructiveness’, ‘failing formality’ and ‘subversion’. Some code groups relevant to the topic are ‘informality/informal practices’, ‘patient organisations' work’ and ‘networks and interactions’. Based on the codes and code groups, several key themes were identified, such as ‘knowledge formatting’, ‘public issue formation’ and ‘interconnected participation practices’. Codes were used for analysing interviews and documents, with both sources of data supplementing each other. In its turn, participatory observations allowed us to reach the level of practices and verify discourses identified in interviews and documents. Lastly, follow‐up interviews were used to triangulate data produced during the observations.

### 2.4 Public Involvement

The initial theoretical framework concerned the topic of informal practices of public participation in nondemocratic conditions. However, we actively sought research participants' perspectives to make our study relevant and relatable to them. As a result, we shifted our focus towards informal tactics by patients of making their interests taken into account by state officials. We also conducted eight repeated interviews to ensure a better fit of theoretical results with the practical needs of the patient community in Russia. Lastly, this article is co‐authored with the patient organisation representative, who has contributed directly to data analysis and presentation, allowing the research team to ensure that a patient's perspective remains in place.

## 3 Results

In this section, we describe numerous uninvited practices that turned out to be widespread within and around invited participation formats. Before doing so, we briefly review the historical development and current state of patient participation in Russia. The review details a network of invited patient participation deliberation formats and their main constituting elements. Notably, some Russian deliberation formats are publics‐created and, therefore, were initially uninvited [26, pp. 52–54]. However, currently, all deliberative formats are either created by the state or state‐sanctioned and, therefore, are invited participation examples.

### 3.1 Invited Patient Participation in Russia

In accordance with historical accounts shared by the Russian patient movement veterans, including Yan Vlasov, before 2006, most patient participation practices, such as one‐on‐one meetings with state officials, were sporadic and informal. One of the few regular participation formats were meetings between patients and state officials in the House of Journalists, with topics reflecting recent publications in the mass media. In 2006, Presidential Order No. 842 established public councils as non‐binding deliberation spaces. The format proved to be a key element of invited patient participation ecosystem in Russia. The Order followed Federal Law 32 (Article 20.2), which, in 2005, established the main regime‐affiliated public participation format, namely, public chambers (Rus. *obschestvennye palaty*) [27].

Since then, unregulated patient participation and separate regional initiatives, such as the Councils on Disability Affairs (Rus. *sovety po delam invalidov*), affiliated with governors' offices, have been gradually substituted by these federal‐level efforts to standardise public participation. This development culminated with the issue of Federal Law 212 ‘On public control’ (2014), which put together fragmentary policy and legislative initiatives on public participation into the framework of a ‘public control’ (FL212, Article 4) [22]. In the Russian legislative framework, public control means a quality of governance oversight by citizens through reporting policy failures or direct misconduct. Public debates over new legislative and policy initiatives, also introduced by the FL212 [22] and a linked Presidential Order No. 601 [28], are legally non‐binding, meaning that nondemocratic state actors retain decision‐making powers in health governance. Although decision‐making powers were not given to publics and even followed by tighter control over uninvited subversive participation practices, such as street protests, public control policies do provide wider opportunities for patients to engage in healthcare governance than the situation before their creation allowed.

**Figure 1:**
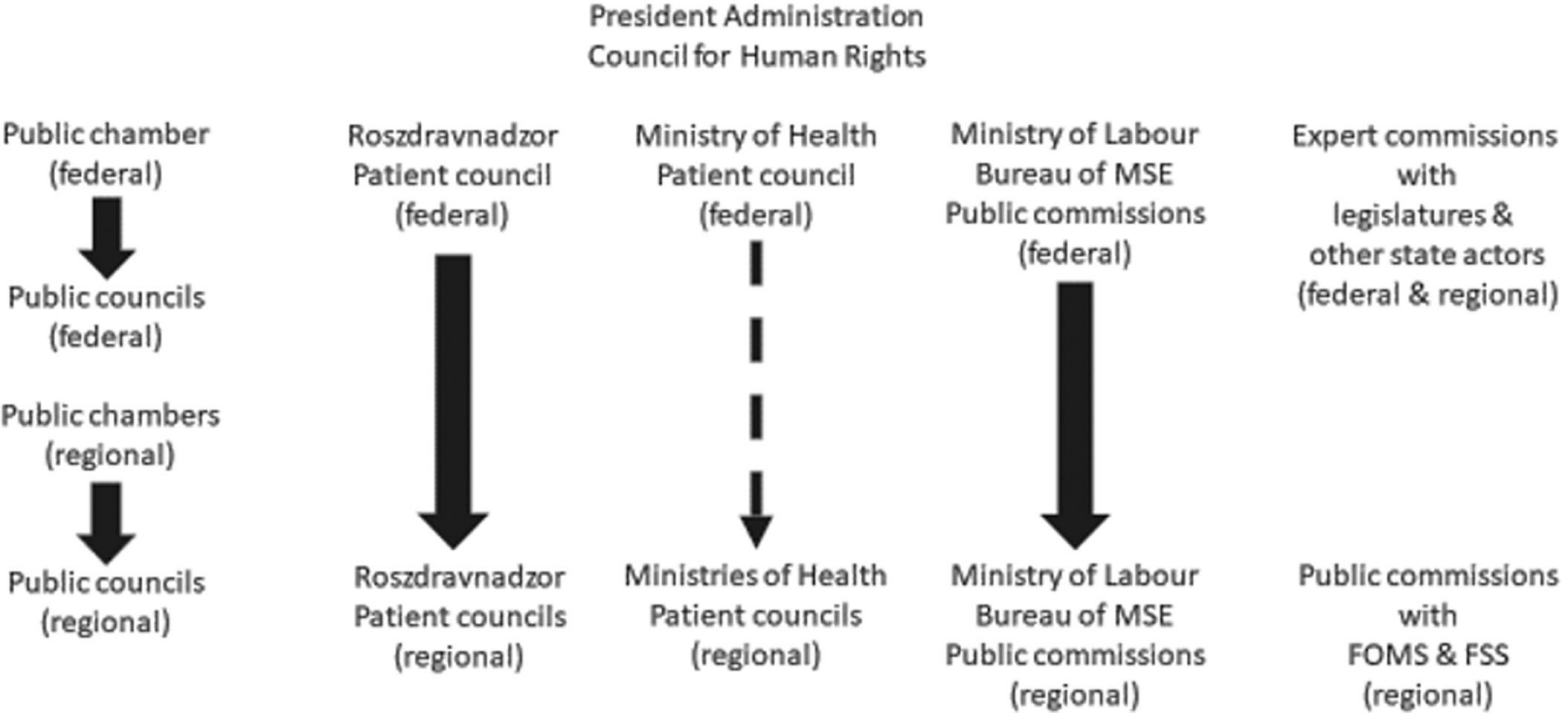
Deliberation in the Russian Healthcare System.

The overall patient participation system in Russia is presented in Figure 1.

Key deliberation formats of those visible in the figure are expert commissions and working groups affiliated with the federal legislatures, public chambers and the Councils for Human Rights and Disability Affairs with the Presidential Administration. The latter two platforms provide patient organisations with access to the highest level decision‐makers. However, of particular importance for patients are public councils, patient councils and MSE public commissions. By law, every state body must have a public council (FL212, Article 13) [22]. These councils take the form of regular—several times a year—meetings of their members, who are usually credentialed experts or prominent public figures, such as scientists or industry representatives. All public council members are invited and approved by public chambers, whose members themselves are appointed by state actors. Public council meetings take place on the premises of a state actor to which they are attached. When asked during the interviews, research participants often referred to the public councils as an ‘imitation’ of participation:

[When public councils were created], state administration structures came in to ensure control over them (…) documents [limiting the independence of public councils] emerged. [A call] that ‘let public councils control state administration’ is [merely] an internal tug war between state actors. (…) In practice, they do not have enough competencies to actually control state actors. Therefore, their work is an imitation [of public participation].

—Igor Garikov, the federal‐level patient council member, author of methodological handbooks on patient participation in Russia

According to Igor Garikov, many public councils are merely tools for internal conflicts between different state actors. The opinion that public councils do not allow for meaningful patient contributions to health governance was corroborated by many other research participants. Observed meetings of the public council with the federal Roszdravnadzor were indeed notoriously dominated by state officials and, at best, credentialed experts rather than citizens.

Another deliberation format was developed by the initiative of patient organisations: patient councils. These councils were developed partly in response to the dominance of credentialed experts in public councils. The first patient council was established by the initiative of major patient organisations in 2006 with the federal Roszdravnadzor office. The Federal Ministry of Healthcare patient council was created in 2012. At about the same time, both Roszdravnadzor (2011–2012) and the Ministry of Healthcare (2012–2013) recommended establishing patient councils at the regional levels. Patient councils were not regulated by federal legislation; thus, each healthcare‐related state institution decided individually whether to create a patient council. Patient councils are an example of invited participation, as members are approved by the state actors to which respective patient councils are attached, meetings are held at the premises of these state institutions, the agenda is coordinated and approved by the state officials and regular meetings are highly organised and official in their status. Unlike public councils, however, the majority of the patient councils' members are patient organisations' representatives.

The third deliberation format crucial for health governance is public commissions affiliated with the Ministry of Labour and Social Affairs MSE bureaus. The first such commission was created in the Genessarea region in 2013. However, many commissions were created later, in 2017–2018, and the federal MSE public commission was established in 2021. The main goal of the MSE public commissions is to oversee the work of MSE bureaus and assist them in determining the disability status of the citizens. This is important for the patient community, as disability status is a condition for various benefits, such as state‐covered rehabilitation. As patient councils, MSE public commissions were created by the initiative of individual MSE bureaus. The standards and procedures for participation in the commission are still under development. For example, Vlas Nikulkin observed a roundtable discussion by patient organisations, in which a dozen of participants actively debated MSE public commission format procedures to be established in the future.

Russian deliberation formats are, in many ways, similar to invited public participation formats in other states. They suffer from the expertisation of patients; venues are in physical proximity to state actors rather than affected communities, and bureaucratic procedures ‘burden’ participating citizens [14, pp. 53–54]. One key difference in the Russian case is the discursive dominance of the state administration over credentialed experts. Furthermore, deliberation in Russia is very precarious, as proven by widely reported postponements and cancellations of meetings during the COVID‐19 pandemic:

Yes, the number of meetings was reduced and the work, let's put it this way, slowed down.

—Dina Grigoryeva, Ivnyak Roszdravnadzor, regional patient council member

Authorities in several regions gladly used the situation [with the pandemic] to close down councils. There is nothing we can do*[about it]*.

—Aleksandr Korepanov, Federal Ministry of Health, patient council member

Overall, the Russian public control framework and the general limitations associated with invited participation might give the impression that independent patient participation is very limited. Nonetheless, patient organisations regularly contributed to health governance. Some notorious examples are amendments to FL 61 related to medical product development procedures, medication provision to rare disease patients by the ‘14 high‐cost nosologies’ programme and incorporation of patient participation in a list of rights of patients in FL 323 (Article 28) [23]. It appeared that these successes depended on uninvited participation situations in what seemed to be invited deliberations. In the next section, we present and discuss multiple examples of such interconnectedness of invited and uninvited participation.

#### 3.1.1 Uninvited Participation Situations in Invited Collaborative Spaces

Uninvited participation has traditionally been studied in terms of either organised collective action, such as protests, or more mundane informal practices, both distinct from invited participation formats [14]. Throughout the fieldwork, however, we encountered many uninvited participation situations in or related to invited participation formats. These situations can be separated into three different categories: (1) situations outside the meetings facilitating work in deliberative bodies, (2) codified tactics for participating in consultative spaces and (3) everyday interactions.

#### 3.1.2 Beyond Collaborative Spaces

Deliberative bodies are a crucial element of wider work on public issue formation [29, 30, 31]. Therefore, patient participation associated with patient and public councils is not limited to setting agendas, holding meetings and publishing minutes on the website. For example, the federal‐level patient organisations manage a string of strategically preplanned conferences and roundtables to which credentialed experts and state officials were invited:

The event was rather official. There was strict moderation of the sessions. Everyone was in official attire. It looked like a ministerial or some other official state meeting. Several speakers represent the federal Ministry of Health. They all knew my research participants and spoke with them as equals. The language used was biomedicine, state administration, and project management. It seemed that during the breaks, patient organisations' leaders met with officials and discussed matters.

—Observation diary: A summary of the large patient community conference in November 2021

The conference in question was attended by state actors up to the federal government level, and various credentialed experts, such as professors, prominent medical professionals and pharmaceutical industry representatives. Interactions and practices at the conference were mostly official. Nonetheless, this was a patients‐led event, as patients set the agenda, invited state actors and credentialed experts and led the discussions.

Among other things, such conferences serve patients' goal of ‘making allies’ from credentialed experts and decision‐makers. Such allies are asked later to support patients in presenting their concerns at the invited participation meetings, either in writing or in person:

‘We prepared materials in support [of our understanding] of the problem. We also sent a letter signed by the director of a national research institute. *(…) [Another research institute's]* department's director made a presentation at the meeting with the federal MSE office director (Irina Germogen, the head of the national‐level patient organisation, the federal patient council member)*.*’ In their work beyond invited deliberative bodies, research participants mentioned uninvited situations, such as writing an email to the regional governor, inviting experts to join a working group, coordinated writing of multiple complaint letters and conducting surveys of their members. Complaints helped document problems, making them tangible to state actors. Survey data were a respected quantitative argument for state actors. Notably, these situations that appeared in preparation for the deliberative councils' meetings were not invited and controlled by the state. Nonetheless, they directly contributed to the agenda setting, discussions at the meetings and the content of minutes.

#### 3.1.3 Uninvited Tactics in Invited Deliberation Formats

Certain patient participation situations proved to be more successful than others in bending and shaping invited deliberations in favour of patients' interests and needs. Patient organisations tracked, summarised and shared these situations with other third‐sector organisations. Many of these situations were uninvited, as they were not part of the institutional arrangements, such as orders of the patient councils, were not sanctioned by the state actors, and defied the public control framework in the work of public issue formation. Drawing on De Certeau, we call these situations ‘tactics’ ([32], p. xiii).

Some of the most widely reported uninvited tactics are ‘allocating additional time before and after the council meetings for informal conversations’:

Before and after the council meeting, people always are there for coffee, tea, conversation.

—Klim Izuzov, regional patient organisation leader, federal patient council member

*[After the end of the official part of the meeting]*. The atmosphere became more ‘friendly’ and ‘informal’. The meeting participants discussed personal matters. The[n] the first [formal] topic to discuss further was palliative care …

—Observation diary entry by a contracted researcher, Syznovo regional Roszdravnadzor patient council meeting, December 2022

Additional time was used either to discuss matters that were not part of the meeting agenda or to elaborate on topics discussed during the meeting. Neither pre‐meeting nor post‐meeting interactions are mentioned in any official document regarding patients or public council organisations. Nonetheless, deliberative bodies' members and state actors present at the meeting readily interact before and after official meetings. According to many experienced patient organisations' representatives, these additional discussions are vital for the inclusion of the patients' concerns into the state policymaking agenda.

Another common observed tactic is ‘substantialising’. Any recommendation, discussion or preliminary agreement at the patient council meetings becomes tangible to the state administration only after being included in the minutes or some other written documents:

*Vlas Nikulkin*: How to control that [non‐binding council's] decision is being taken into account and included in the decision‐making?

*Ivan Korepanov, the federal patient council member*: First, it is necessary to have the decision included in the minutes. Then, what is called the realisation of the recorded decision begins. By referring to the minutes, [patient organisations] work with state institutions. Another great mechanism is to review minutes and record decisions at the end of the year. Remind heads of the state institutions about them and ask to make an order to implement the decision.

Writing decisions down in some official documents and using additional time for informal conversations were just two tactics out of several dozen mentioned in the methodological documents (e.g., Doc 44 ‘Public council engagement technologies’; Doc 55 ‘Handbook on public control practices’; Doc 58 ‘Best practices of state engagement’) and identified during our fieldwork. These tactics are a product of years of experience with patients engaging in the Russian state. They facilitated issue formation by patient organisations in deliberative bodies that were not intended and encouraged by Russian state actors. In other words, uninvited participation situations described as tactics appeared regularly in the state‐created deliberation formats, despite the overall dominance of invited participation situations.

#### 3.1.4 Everyday Practices

The tactics that we observed were practices that were well reflected, documented and standardised. However, we also encountered many everyday practices that accounted for situational and mundane ‘playing the system’ [14, p. 101] situations. Such situations were particularly common in working groups created in public and patient councils. Usually created ad hoc, without any particular formal procedures involved, working groups are very shifty in their practices and content. Some of them are held in cafes:

They *[members]* would meet in a cafe, without me *[a chair]* (…) The last several years, we do not make it formal, just discuss things—as I call it later in the minutes—‘in a form of the working group’.

—Anatoly Nadtochy, the regional patient council member

Others take the form of tea‐drinking parties:

We drink tea, eat doughnuts, and chat. It is another level of interaction (…) We meet at her's *[patient council member's working office]*.

—Domna Domna, the regional patient council member

Meetings in a cafe, chats in WhatsApp and tea drinking are all not situations invited by the state and are far from institutional decision‐making, as state officials are rarely present at the working group meetings. Nonetheless, working groups are inseparable and a crucial part of invited participation. Moreover, according to Domna Domna, the meetings allowed for discussing ‘the most pressing problems’, which would otherwise remain unaddressed and, therefore, silenced during the invited participation situations.

Other everyday, uninvited situations appear at an open microphone part of the meeting, called ‘miscellaneous’ (Rus. *raznoe*). After agenda points are discussed, the patient councils' chairs usually ask all present for additional topics in mind. Several research participants mentioned that, at that moment, unapproved complaints, problem definitions and potential solutions might be presented to state actors and credentialed experts:

Topics that are raised in ‘miscellaneous’ might be of more importance than those in the agenda. They might be very thorny, and they might be framed and discussed in various ways [unlike topics in the agenda, which are predefined and thoroughly negotiated with the state actors]

—Klim Izuzov, regional patient organisation leader, the federal patient council member

The patient organisations make use of the uncertainty of the ‘miscellaneous’ topics:

[Miscellaneous] is the most unpredictable [part of the meeting]. The topic is brought in, but how the discussion will turn up is unknown. Patients can bend it in their favour.

—Klim Izuzov, regional patient organisation leader, federal patient council member

‘Miscellaneous’ are a part of the official patient council meetings' deliberations and, therefore, members *are invited* to contribute unsolicited complaints and questions. However, what and how is being discussed depended on very mundane things, such as who could speak louder, or have a wider network of supporters.

Moreover, miscellaneous topics, even if not included in policymaking immediately, still appeared in a public debate by being written down in the minutes. This also means that invited participation situations, such as writing minutes, become intertwined with uninvited situations, such as being able to scream or being able to pitch the problem in a concise and convincing way. Notably, speaking in a louder voice, talking to other members before the meeting, drinking tea and chatting during working group meetings were just a few of multiple everyday uninvited participation situations identified during our fieldwork.

## 4 Discussion

Regarded ‘institutionally linked to policy making’ and ‘preordinated’ [11, p. 107], invited participation implies that it is sanctioned and controlled by state actors or credentialed experts. In this framework, state–citizens' interactions, such as citizen assemblies, public hearings and, in our case, patient and public councils, limit the independence of citizens and help cement the dominance of expert‐driven discourses [11, p. 107]. Despite this widely accepted assumption, Russian patient organisations regularly use state‐created deliberative bodies for public issue formation [30, 31]. They do so, among other things, with the help of uninvited situations, whether they work beyond meetings, tactics or multiple everyday interactions. All these uninvited practices, in one way or another, challenge ‘implicitly imposed normative commitments—an implicit politics—as to what is salient and what is not salient’ [11, p. 107]. It also means that patient participation practices are interconnected and interdependent [33], creating a specific participation ‘milieu’ [34, p. 195] rather than clearly defined separate instances of engaging decision‐makers.

We provided multiple examples of situations that are neither institutionally linked to policy‐making, nor completely fit into the normative commitments of the Russian state actors. However, these uninvited practices are an inseparable and crucial part of non‐binding deliberation formats, such as patient councils. As a result, Russian deliberative bodies do not only maintain social stability maintenance and a dominance of state administration but also the interests, issues and practices of patient organisations not coordinated with the state or credentialed experts. Therefore, these spaces are not uniform and cannot be described as exclusively ‘invited’ or ‘uninvited’. Instead, each deliberative interaction should be viewed as an ecology of actors and practices rather than uniform power relations [35, p. 26]. Moreover, different practices might support and reinforce each other: public control opens opportunities for public issue formation, while public issue formation helps maintain social stability.

Crucially, such interconnectedness of invited and uninvited participation is not a property of the Russian case only. Health provision and organisation in the United Kingdom [14], food production in the Global South [36] or expansion of participatory governance in the Netherlands due to the changed ‘planning culture’ [37] all involve an ecology of actors and practices allowing for participation and contributions to governance disregarding unequal power dynamics or limited formal access to decision‐making. The concept of ‘situations’ [18, p. 18], which implies singularity and multiplicity, helps explain how uninvited practices appear in the *predominantly* invited settings.

## 5 Conclusion

In this article, we presented the case of Russian deliberative bodies, which are being appropriated and used by patient organisations for the interests of their beneficiaries. We conceptualised these state‐created bodies as invited participation examples. At the same time, we discussed a multiplicity of uninvited participation practices widely co‐presented in the very same deliberation situations. As a result, we were able to deconstruct a binary approach to invited and uninvited participation as unviable. Instead, the ecologising approach, which interprets invited and uninvited practices as interconnected, has better explanatory power for cases in which citizens maintain independence and issues are being formed despite all limitations associated with invited participation or even authoritarian settings. We further developed the ecologising approach by conceptualising invited and uninvited practices as situations, or separate time‐ and space‐bound singularities. By treating participation as such separate singularities, we explain how different juxtapositions of power emerge in the seemingly unequal deliberation between patients and state actors, which are supposed to be completely invited and, therefore, dominated by credentialed experts and decision‐makers.

Direct implications of the ecologising approach for patient and public involvement practice are that the patient community should pay more attention to everyday practices and be aware of informal tactics. Specific recommendations for policymakers and patients alike include embracing and investing in maintaining informal relations they build, ensuring that the organisation of participatory engagements allows space for improvisation and including vocational training of patients in applying informal tactics proven successful for engaging decision‐makers in specific (industry, country and participation format) settings. Noteworthy, more detailed practical advice and theoretical considerations related to ecologising approach have to be developed by additional studies, which would involve decision‐makers, who were not participating in our research. The applicability of fluidity to more predictable, democratic settings, in which powerholders do not have such excessive powers also needs to be further investigated.

## Author Contributions

**Vlas Nikulkin:** conceptualisation, investigation, writing–original draft, methodology, writing–review and editing, project administration, formal analysis, validation, data curation. **Yan Vlasov:** writing–original draft, validation, writing–review and editing, formal analysis, conceptualisation. **Olga Zvonareva:** conceptualisation, methodology, supervision, project administration, writing–review and editing, resources, validation, funding acquisition.

## Data Availability

Data contain qualitative sensitive personal information, are kept in a protected external drive and, in accordance with the consent forms and the decisions by Maastricht University Faculty of Health, Medicine and Life Sciences Research Ethical Committee FHMLREC/2021/109 and Saint Petersburg Association of Sociologists—SPAS 03.12.2021 No. 1), are not to be shared.
